# Beyond the Tropics: Clinical Presentation and Epidemiology of Travel-Associated Dengue Fever in a Non-Endemic European Setting

**DOI:** 10.3390/microorganisms14071439

**Published:** 2026-06-30

**Authors:** Lena Dollensky, Hermann Laferl, Cristina Groza, Christoph Wenisch, Alexander Zoufaly, Tamara Clodi-Seitz

**Affiliations:** 1 Medical Faculty, Sigmund Freud Private University, 1020 Vienna, Austria; lena.dollensky@gmail.com (L.D.); alexander.zoufaly@mail.sfu.ac.at (A.Z.); 2 Department of Infectious Diseases and Tropical Medicine, Clinic Favoriten, 1100 Vienna, Austria; hermann.laferl@gesundheitsverbund.at (H.L.); cristina.groza@gesundheitsverbund.at (C.G.); christoph.wenisch@gesundheitsverbund.at (C.W.)

**Keywords:** dengue, dengue with warning signs, fever in returning travellers

## Abstract

Dengue is a rapidly spreading mosquito-borne viral disease with increasing incidence in Europe, driven by international travel and climate-related expansion of Aedes mosquito habitats. Data on imported dengue in Central Europe remain limited. The aim of this study was to characterize clinical and epidemiological features of imported dengue infections in Austria and identify factors associated with dengue warning signs. A retrospective, single-centre observational study conducted from January 2013 to October 2024. We retrospectively analyzed 175 adults with confirmed travel-associated dengue treated in Vienna, Austria. Demographics, travel history, symptoms, laboratory values, and clinical outcomes were extracted from electronic records. WHO criteria were applied to classify dengue severity. Most infections were acquired in Southeast Asia (72.6%), particularly Thailand. Common symptoms were fever (97.1%), fatigue (97.1%), arthralgia (59.4%), and rash (53.8%). Dengue warning signs were observed in 16% of cases and associated with significantly prolonged leukopenia (*p* = 0.003), increased hospitalization rates (50.0% vs. 23.8%, *p* = 0.005), and higher frequencies of vomiting, abdominal pain, mucosal bleeding, and petechiae at first medical contact. Women reported rash, arthralgia, and retro-orbital pain more frequently, while men showed lower platelet counts. No cases of severe dengue or deaths occurred. Dengue should be considered in febrile returning travellers, particularly from Southeast Asia. The presence of warning signs predicts more complicated disease courses and should prompt closer clinical monitoring. These findings underscore the importance of awareness and preparedness for dengue in non-endemic European settings.

## 1. Introduction

Dengue is a vector-borne viral infection transmitted mainly by Aedes mosquitoes, particularly Aedes aegypti and Aedes albopictus [1]. There are four closely related but serologically distinct serotypes of dengue virus (DENV), all of which belong to the flavivirus genus: DENV-1, DENV-2, DENV-3 and DENV-4 [2].

Globally, approximately 400 million people are infected with dengue each year, of whom about 100 million develop symptomatic disease. An estimated 40,000 deaths occur annually, corresponding to a mortality rate of approximately 0.01% among infected individuals [3]. The clinical picture of dengue fever varies greatly. The incubation period is between 3 and 14 days [4]. In some cases severe forms occur which can take a life-threatening course. In these cases patients may suffer from hemorrhagic diathesis, capillary leakage, shock, and multi-organ failure [5].

In recent years, a sharp increase in dengue cases has been observed. While the majority of infections still occur in tropical and subtropical regions—where large outbreaks continue to occur—Europe has also seen a growing number of cases [3].

Due to climate change, Aedes mosquito species have become established in parts of Europe, creating suitable conditions for local transmission [6].

Aedes albopictus is now established in 28 European countries [7,8]. Due to its remarkable ability to adapt to new environmental conditions, it is considered one of the most invasive mosquito species in the world [9,10]. The first reported occurrence in Europe was in Albania in the 1970s. In the following decades, the mosquito continued to spread to Italy (1990), France (1999), Belgium (2000), Spain (2003), Switzerland (2003), Croatia (2004) and The Netherlands (2005) [11]. Forecasts indicate a further shift of Aedes albopictus to Central Europe, while its presence in the Mediterranean region is expected to decline due to increasingly dry summer conditions [9,10,12,13]. While Aedes aegypti is primarily found in tropical and subtropical regions, its reappearance in Europe was recently reported in Madeira, Portugal, in 2012 [14].

The increase in international travel further facilitates the spread of the dengue virus in Europe [15,16]. As a large proportion of infections is 40–80% [3,17], the introduction of the virus often goes unnoticed. The greatest risk is when viraemic individuals return during the European summer, a time when Aedes mosquito activity is at its highest, creating favourable conditions for local transmission and possible outbreaks in Europe [18]. As an example, in 2020, the first five autochthonous cases of dengue virus type 1 in Italy were identified as a family cluster in the province of Vicenza, a region where Aedes albopictus is endemic. The outbreak was triggered by a traveller returning from Indonesia. The outbreak was triggered by travel returnee from Indonesia [14].

These developments underline the fact that dengue fever is no longer restricted to tropical regions but is increasingly becoming a public health problem in Europe. As autochthonous transmission becomes more likely, not only travel health and infectious disease specialists need to be sensitized, but also general practitioners and specialists in internal medicine, who are often the first point of contact for patients.

To increase knowledge of travel-associated dengue fever, a retrospective analysis of infected Austrian tropical returnees was conducted with the aim of describing the clinical and epidemiological characteristics of this population. Improved awareness and faster detection can support earlier diagnosis and appropriate treatment, reducing the risk of severe outcomes and limiting the potential for onward transmission.

## 2. Methods

### 2.1. Aim

The primary aim of this study is to describe the proportion of returning travellers who have been diagnosed with dengue in Austria in the last 12 years. The study focuses, in particular, on travel patterns, clinical symptoms and laboratory findings at first presentation as well as the clinical course of the disease. In addition, possible factors associated with the course of the disease and gender-specific differences are analyzed.

### 2.2. Study Population

This retrospective analysis included all patients with imported, travel-associated, confirmed dengue infections treated at the Department of Infectious Diseases and Tropical Medicine at the Clinic Favoriten in Vienna, Austria, from January 2013 to October 2024. Inclusion criteria were age over 18 years, a confirmed dengue infection and a complete medical history. Patients under the age of 18 or with insufficient clinical data were excluded. The study was approved by the Ethics Committee of the City of Vienna (EK23-197-VK) and the Sigmund Freud Private University of Vienna (EK 998-2024).

### 2.3. Data Extraction

The data were collected retrospectively using the electronic patient documentation system at the Department of Infectious Diseases and Tropical Medicine at the Clinic Favoriten in Vienna, Austria. The identified cases were registered, and their medical records were then reviewed in detail for data collection.

### 2.4. Definitions

According to the US Centers for Disease Control and Prevention (CDC), a confirmed dengue case is defined as a clinically compatible illness with laboratory evidence of infection (detection of dengue virus RNA by PCR assay, detection of non-structural protein 1 antigen, or a fourfold or greater increase or decrease in IgG and/or IgM antibody titers in paired acute and convalescent serum samples, as determined by enzyme-linked immunosorbent assay or equivalent serologic methods) [3,19,20]. In our centre depending on the timing of presentation, diagnosis was based on a combination of molecular testing, NS1 antigen detection, and/or dengue-specific serology, in accordance with current diagnostic recommendations. Potential cross-reactivity with other flaviviruses is a recognized limitation of serological testing. In routine clinical practice, molecular assays and antigen detection methods are preferred during the acute phase of infection. In cases diagnosed by serology, results were interpreted in conjunction with clinical presentation, travel history, and, where indicated, additional confirmatory testing. Other arboviral and flaviviral infections, including Zika virus, chikungunya virus, yellow fever virus, and Japanese encephalitis virus, were considered as part of the differential diagnostic work-up when clinically appropriate.

A probable case is defined as a clinically compatible illness in a person who has recently travelled to or stayed in a dengue-endemic area. In these cases, IgM seropositivity supports the diagnosis, but is not sufficient to confirm it in the case of possible co-exposure to other flaviviruses [3].

Dengue infections are divided into dengue without warning signs and dengue with warning signs, depending on the presence or absence of clinical and laboratory features that indicate an increased risk of severe disease [3,17].

Dengue with warning signs is characterized by the presence of clinical criteria for dengue infection in conjunction with additional findings that indicate an increased risk of developing severe disease. These warning signs include abdominal pain or tenderness, persistent vomiting, clinical signs of fluid accumulation (such as ascites or pleural effusion), mucosal bleeding, marked lethargy or restlessness, liver enlargement greater than 2 cm, or a concomitant increase in hematocrit with a rapid decrease in platelet count [17,18,21].

Severe dengue fever is defined by the presence of one or more of the following symptoms: capillary leak syndrome—characterized by plasma leakage due to increased vascular permeability—severe bleeding or organ dysfunction. The latter may be indicated by transaminase levels (AST or ALT) > 1000 IU/L, neurological symptoms with impaired consciousness and severe organ impairment [3,17].

### 2.5. Statistics

Data analysis was performed with IBM SPSS Statistics (version 29.0.2.0 (20)). Normally distributed variables are presented as mean ± standard deviation (SD), while non-normally distributed variables are summarized as medians with interquartile ranges (IQR).

Categorical variables are presented as absolute frequencies and percentages. Group comparisons between patients with and without dengue warning signs were performed using the chi-square test for independence or Fisher’s exact test if the expected cell counts were less than five. For continuous variables, the *t*-test was used for normally distributed data and the Mann–Whitney U test for non-normally distributed data.

Statistical significance was defined as a two-sided *p*-value < 0.05.

## 3. Results

### 3.1. Epidemiology

**Figure 1:**
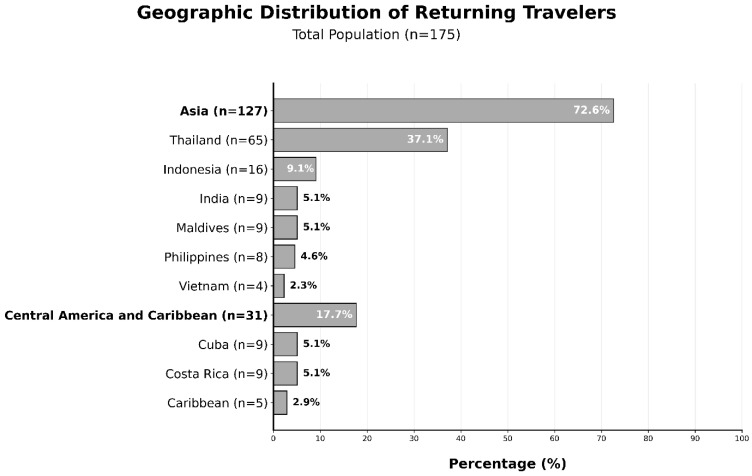
Distribution of travel-associated dengue cases by continent of travel origin.

This retrospective analysis included 175 patients. All included patients met the CDC criteria for confirmed dengue infection. As shown in Figure 1, the majority of dengue cases were acquired in Asia, particularly Southeast Asia, followed by Central America.

**Figure 2:**
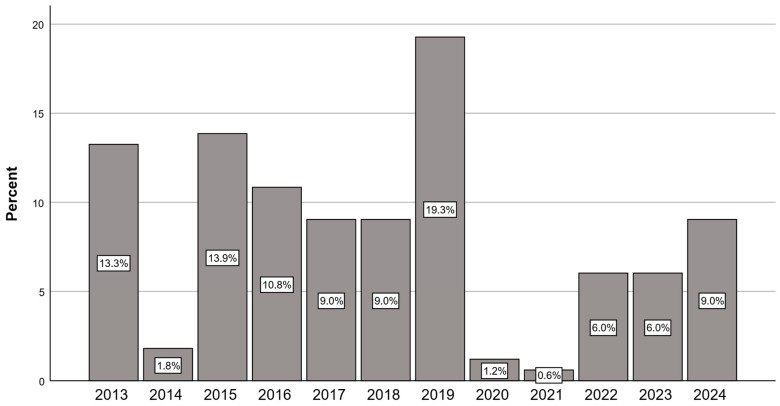
Annual distribution of travel-associated dengue cases among returning travellers to Austria, 2013–2024.

The highest number of dengue cases among returning travellers in our centre was recorded in 2019, followed by a significant decrease during the COVID-19 pandemic years (2020–2022), as shown in Figure 2.

**Figure 3:**
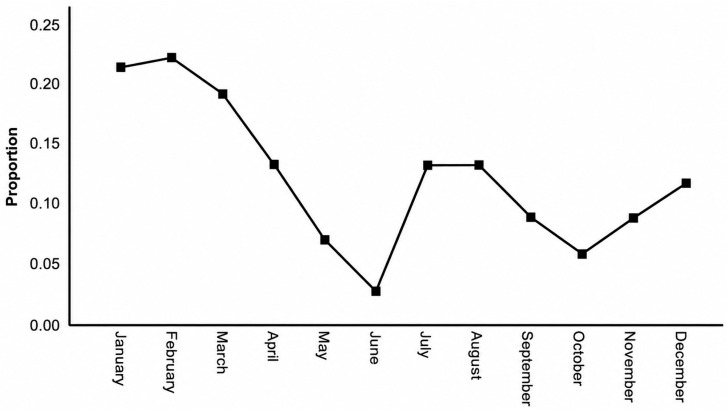
Monthly distribution of imported dengue cases.

A more detailed analysis of the months shows that the majority of cases occurred in February followed by January, as shown in Figure 3.

### 3.2. Baseline Characteristics

A total of 175 patients were included in the study. The average age was 41.2 years (±SD 13.6) and 48.6% were female.

### 3.3. Clinical Presentation

**Figure 4:**
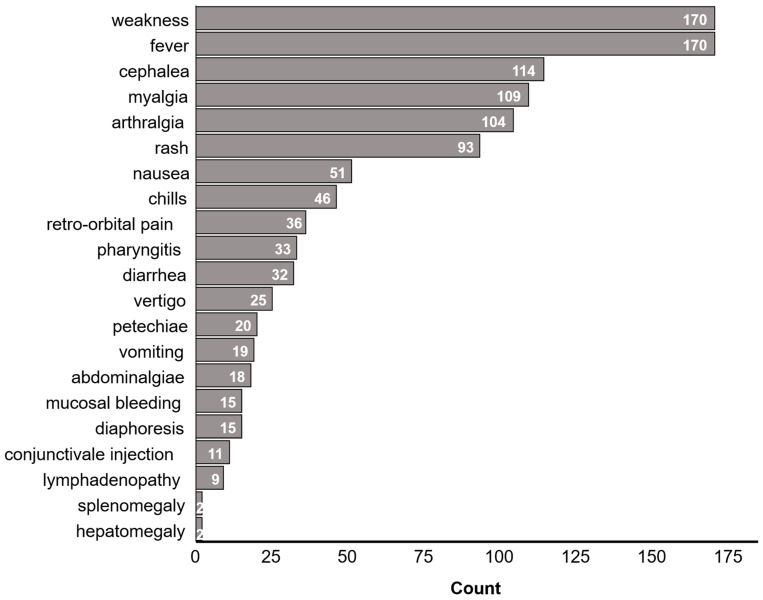
Reported symptoms and their frequency among dengue patients.

As seen in Figure 4, the most common symptoms at the time of initial presentation were fever and weakness (97.1%) Other symptoms included retro-orbital pain, headache, arthralgia, myalgia, nausea, diarrhea and vomiting. In addition, 53.8% of patients showed a skin rash. Petechiae occurred in 11.4% of all patients with dengue. In 49.1% of patients, symptoms lasted longer than seven days. Women developed arthralgia (♀ 67.1%, ♂ 52.2%, *p* = 0.046), skin rash (♀ 61.9%, ♂ 46.1%, *p* = 0.037) and retro-orbital pain (♀ 29.4%, ♂ 12.2%, *p* = 0.005) significantly more frequently than men.

### 3.4. Laboratory Findings

At the time of initial presentation, thrombocytopenia occurred in 74.9% of cases and leukopenia in 70.3%. Elevated liver enzymes were observed in 81.1%. Thrombocytopenia was observed more frequently in male patients (♀ 67.1%, ♂ 82.2%, *p* = 0.021). In addition, the median platelet count at first presentation was significantly lower in men than in women (Md ♀ 142 G/L ♂ 117 G/L, *p* = 0.004).

If thrombocytopenia or leukopenia were present, they lasted longer than a week in 13.1% and 9.2% of all cases. Elevated liver enzymes were observed longer than 7 days in 32.5%. Thrombocytopenia persisted longer than seven days more often in male individuals (♀ 8.5%, ♂ 17.6%, *p* = 0.042).

### 3.5. Clinical Outcomes

Forty-nine patients (28.0%) had to be admitted to hospital. The main reasons for hospitalization were severe thrombocytopenia and/or bleeding diathesis or significantly worsened general condition. The average length of hospitalization was 4.5 days (min-max 2–7, SD +/−1.4).

Twenty-eight patients (16%) showed dengue warning signs, in particular mucosal bleeding, especially menorrhagia and epistaxis, as well as severe abdominal pain.

No severe dengue cases were recorded. One admission to the Intermediate Care Unit was documented, the transfer was initiated due to severe thrombocytopenia. Mortality rate was zero.

### 3.6. Factors Associated with Warning Signs

In a cohort of 175 patients with confirmed dengue infection, 28 patients (16%) had warning signs. Female patients were more frequently represented in the group with warning signs (60.7% vs. 46.3%), although this difference did not reach statistical significance (*p* = 0.161). There were no significant differences shown between patients presenting with warning signs compared to these without warning signs regarding basic parameters, like age, presence of comorbidities and BMI. No differences were shown regarding travel-associated illness, like duration from onset symptoms to first clinic visit, duration of travel, region of exposure and reason of travel.

Patients with dengue with warning signs presented as significant more often with nausea (*p* = 0.008), diarrhea (*p* = 0.009), vomiting (*p* = 0.016), abdominal pain (*p* < 0.001), petechiae (*p* = 0.021) and mucosal bleeding (*p* < 0.001) at time of first visit to clinic. No significant difference was observed regarding other symptoms or laboratory abnormalities at time of first visit. If leukopenia was present, patients with dengue with warning signs presented with longer duration of leukopenia (*p* = 0.003). There was a trend to prolonged thrombocytopenia and risk of dengue with warning signs, but it was not statistically significant. There was no statistically significant difference seen between duration of thrombocytopenia or elevation of hepatic enzymes or fever. Patients with dengue with warning signs were admitted to hospital significantly more often (*p* = 0.005). The exact data are shown in Table S1 in the Supplementary File.

## 4. Discussion

This retrospective analysis highlights important clinical and epidemiological characteristics of imported dengue cases in Vienna, Austria, over a 12-year period. Among returning travellers in the whole of Austria, a total of 1100 travel-associated dengue cases were identified from 2013 to 2024. Between 2013 and 2019, a continuous year-on-year increase in dengue cases was observed. This trend was interrupted by a pronounced decrease during the COVID-19 pandemic in 2020–2022, after which case numbers increased again, culminating in a peak of 203 cases in 2024. In our cohort in Vienna, a total of 175 patients with confirmed dengue infection were included. The majority of whom had recently travelled to Southeast Asia, particularly Thailand. This is consistent with international surveillance data, which identify Asia as a key source of imported dengue cases to Europe [13].

Studies show that dengue infections generally rise among travellers returning to Europe, confirming the growing importance of dengue in non-endemic regions over the last decade [22,23,24,25,26]. A marked decline in cases was observed in our cohort during the COVID-19 Pandemic in 2020–2022 [27], as seen in our cohort as well. This trend reflects the impact of international travel restrictions, reduced global mobility, and public health measures implemented during the pandemic, which significantly limited exposure to dengue-endemic regions.

A seasonal pattern emerged, with most cases presenting during winter months (December–February). This correlates with the peak travel periods to tropical regions during European winter. Also, in Austria, most long-distance travel takes place in the first and fourth quarters of the year [28].

At first medical contact, most patients in our cohort presented with fever (97.1%), arthralgia (59.4%) and rash (53.8%), which is in line with previous European studies. A literature review reported fever in nearly all cases, arthralgia occurring in 50–79% and rash in 41–68% of patients [29].

Interestingly, in our study, women reported arthralgia, skin rash and retro-orbital pain significantly more frequently than men. In other European studies, gender-specific differences were not evaluated. In a retrospective Indian study, women with dengue reported arthralgia, myalgia and headache significantly more often than men. The presence of skin rash or retro-orbital pain was not evaluated in the Indian study [30].

Laboratory findings at first medical contact included thrombocytopenia and leukopenia in over 70% of the patients and elevated transaminases in over 80%, consistent with the known pathophysiology of dengue and several other studies [23,24,26,30,31].

Male patients presented significantly more frequently with thrombocytopenia and a lower platelet count at first medical contact than female ones in our cohort. This is in line with a Brazilian study, which also showed that there is a significantly higher number of thrombocytopenia in male patients [32]. However, another study from Pakistan showed no statistically significant variations in platelet counts between male and female patients [33].

Our study demonstrates that individuals with symptomatic dengue can experience prolonged illness. Approximately half of the patients reported symptoms lasting more than one week, and 8.0% even reported symptoms persisting for more than two weeks. The median duration of fever was 5 days, but cases of fever lasting up to 14 days were reported. Laboratory abnormalities may also persist, for example, elevated liver enzymes were detectable for over one week in more than 30% of the cases.

Sixteen percent of the patients in our cohort developed dengue with warning signs. No cases of severe dengue were recorded, and the case fatality was zero. The hospitalization rate was 28%.

Hospitalization was significantly more common in our cohort in patients presenting with warning signs (50.0% vs. 23.8%; *p* = 0.005).

Female patients were more frequently represented in the group with warning signs (60.7% vs. 46.3%), although this difference did not reach statistical significance (*p* = 0.161). No significant differences were found regarding age and comorbidities and the development of dengue with warning signs.

In contrast to our findings, several other studies have identified higher age and pre-existing medical conditions as important determinants of more severe dengue manifestations [34,35]. An explanation may be the rather young median age and lack of severe chronic diseases in our cohort.

Interestingly, leukopenia persisted significantly longer in patients with warning signs. This had already been postulated by authors in an earlier study, which demonstrated that leukocyte counts on day 3 and day 5 after symptom onset had no impact on disease severity. However, the leukocyte count after day 7 showed a statistically significant influence on the severity [36]. In our cohort there was a trend to prolonged thrombocytopenia and risk of dengue with warning signs as well, which was not statistically significant.

As expected, symptoms such as vomiting (25.0% vs. 8.2%), mucosal bleeding (53.6% vs. 0%) and abdominal pain (64.3% vs. 0%) were significantly more common in patients with dengue with warning signs, as these features are part of the WHO case definition [17]. Consequently, their statistical significance primarily reflects definition criteria rather than independent clinical differentiation. Other symptoms such as petechiae (25.0% vs. 8.8%), nausea (50.0% vs. 25.2%) and diarrhea (35.7% vs. 15.0%), which are not part of the formal definition, also occurred significantly more frequently.

### 4.1. Limitations

This study has some limitations that should be considered. First, the retrospective design of the study inherently limits control over the completeness, consistency, and standardization of the data. Important clinical variables may not have been uniformly documented, which may lead to information bias. Second, the study was conducted in a single hospital in Vienna, which may limit the generalizability of the results to broader populations, particularly those from other geographic regions or healthcare systems. Nonetheless, Clinic Favoriten is one of the largest referral centres for tropical medicine in Austria and serves a large catchment area, which increases the representativeness of the sample in a national context. Thirdly, a selection bias cannot be ruled out, as only patients who received treatment at the Department of Infectious Diseases and Tropical Medicine were included. Persons with milder disease courses or those treated as outpatients or in other facilities may be underrepresented, which could lead to an overestimation of the severity of symptoms and hospitalization rates.

Although the total cohort included 175 patients, only 28 of them had warning signs, which limited the statistical power for subgroup analyses and increased the risk of failing to detect true differences or associations due to insufficient sample size. The study also did not include serotype-specific diagnostic tests, limiting the interpretation of serotype-related differences in clinical presentation or disease severity. In addition, categorization into “dengue with warning signs” or “dengue without warning signs” was based on available clinical documentation and the application of WHO criteria; therefore, subtle or undocumented symptoms may have led to misclassification.

Travel history was self-reported, which may lead to recall bias, especially in the case of multiple exposures or stopovers in endemic areas. In addition, no data on previous dengue infections or vaccination status were available, although these could have an impact on clinical severity and immune response.

### 4.2. Conclusions

The potential for local transmission of dengue in Europe presents a growing concern. The increasing presence of competent vectors, such as Aedes albopictus, which is now established in large regions of Southern and Central Europe, and the reappearance of Aedes aegypti in large areas in certain European regions, in combination with viraemic travellers during the active mosquito season, constitutes a credible risk scenario for the occurrence of autochthonous transmission [7,8,11]. The data from our study underscore the frequency of warning signs and hospital admissions, highlighting the clinical burden of imported dengue and the importance of careful outpatient management.

## Data Availability

The datasets generated and/or analyzed during the current study are available from the corresponding author on reasonable request.

## Supplementary Materials

The following supporting information can be downloaded at: https://www.mdpi.com/article/10.3390/microorganisms14071439/s1, Table S1: Comparison between patients with dengue with warning signs and patients with dengue without warning signs.

## Abbreviations

ALT	Alanine aminotransferase
AST	Aspartate aminotransferase
BMI	Body mass index
CDC	Centers for Disease Control and Prevention
CI	Confidence interval
DENV	Dengue virus
IQR	Interquartile range
OR	Odds ratio
PCR	Polymerase chain reaction
SD	Standard deviation
WHO	World Health Organization
