# Supplementary material for: Beyond the Tropics: Clinical Presentation and Epidemiology of Travel-Associated Dengue Fever in a Non-Endemic European Setting
Source: Microorganisms. 2026 Jun 30;14(7):1439. doi: 10.3390/microorganisms14071439 (PMC13413981; doi:10.3390/microorganisms14071439)
Supplement: Supplementary file 1 [file microorganisms-14-01439-s001.zip › microorganisms-4369089-supplementary.pdf]

**Table S1.**: Comparison between patients with dengue with warning signs and patients with dengue without warning signs. *** statistically significant.**

| | **total (n = 175)** | **with warning signs (n = 28)** | **without warning signs (n = 147)** | **p - value** |
|---|---|---|---|---|
| **Sex (n = 175)** | | | | |
| Female | 85 (48.6) | 17 (60.7) | 68 (46.3) | 0.161 |
| **Age (years) (n = 173)** | | | | |
| M ± SD | 41.2 ± 13.6 | 36.5 ± 12.0 | 42.1 ± 13.7 | 0.230 |
| **Illness onset to clinic (days) (n = 172)** | | | | |
| Md (IQR) | 4 (2 – 6) | 3.5 (2 – 5.8) | 4 (2 – 6) | 0.420 |
| Min - Max | 0 – 19 | 0 – 11 | 0 – 19 | |
| **Duration of fever** | | | | |
| Md (IQR) | 5 (3.5 – 6) | 5 (4 - 6) | 5 (3 - 6) | 0.400 |
| Min - Max | 0 - 14 | 1 - 8 | 0 - 14 | |
| **Duration of travel (days)** | | | | |
| (n = 167) | | | | |
| Md (IQR) | 17 (14 – 23) | 16 (13 – 21) | 17 (14 – 23.8) | 0.401 |
| Min - Max | 4 – 273 | 8 – 244 | 4 – 273 | |
| **Region of exposure (n = 175)** | | | | |
| Africa | 10 (5.7) | 2 (7.1) | 8 (5.4) | 0.097 |
| Asia | 127 (72.6) | 17 (60.7) | 110 (74.8) | |
| Central America | 31 (17.7) | 6 (21.4) | 25 (17.0) | |
| Oceania | 2 (1.1) | 0 (0.0) | 2 (1.4) | |
| South America | 5 (2.9) | 3 (10.7) | 2 (1.4) | |
| **Reason for travel (n = 112)** | | | | |
| Tourism | 98 (87.5) | 18 (85.7) | 80 (87.9) | 0.864 |

| | | | | |
|---|---|---|---|---|
| Family / Friends | 9 (8.0) | 2 (9.5) | 7 (7.7) | |
| Business | 5 (4.5) | 1 (4.8) | 4 (4.4) | |
| **Presence of comorbidities** | | | | |
| (n = 175) | 46 (26.3) | 7 (25.0) | 39 (25.3) | 0.866 |
| **BMI Classification** | | | | |
| Underweight | 3 (2.7) | 0 (0.0) | 3 (3.1) | 0.346 |
| Normal | 64 (57.1) | 7 (43.8) | 57 (59.4) | |
| Overweight | 33 (29.5) | 9 (56.3) | 24 (25.0) | |
| Obesity | 12 (10.7) | 0 (0.0) | 12 (12.5) | |
| **Clinical symptoms** | | | | |
| fever (n=175) | 170 (97.1) | 28 (100) | 142 (96.6) | 1.000 |
| weakness (n=155) | 170 (97.1) | 27 (96.4) | 143 (97.3) | 0.586 |
| cephalea (n = 175) | 114 (65.1)) | 17 (60.7) | 97 (66.0) | 0.592 |
| myalgia (n = 175) | 109 (62.3) | 18 (64.3) | 91 (61.9) | 0.812 |
| arthralgia (n = 175) | 104 (59.4) | 18 (64.3) | 86 (58.5) | 0.568 |
| rash (n = 175) | 93 (53.8) | 18 (64.3) | 75 (51.7.) | 0.222 |
| nausea (n = 175) | 51 (29.1) | 14 (50.0) | 37 (25.2) | **0.008*** |
| chills (n = 166) | 46 (27.7) | 11 (39.3) | 35 (25.4) | 0.133 |
| retro-orbital pain (n = 175) | 36 (20.6) | 9 (32.1.) | 27 (18.4) | 0.098 |
| pharyngitis (n = 175) | 33 (18.9) | 6 (21.4) | 27 (18.4.) | 0.704 |
| diarrhea (n = 175) | 32 (18.3) | 10 (35.7) | 22 (15.0) | **0.009*** |
| vertigo (n = 175) | 25 (14.3) | 2 (7.1) | 23 (15.6) | 0.377 |
| petechiae (n = 175) | 20 (11.4) | 7 (25.0) | 13 (8.8) | **0.021*** |
| mucosal bleeding (n = 175) | 15 (8.6) | 15 (53.6) | 0 | **<0.001*** |

| | | | | |
|---|---|---|---|---|
| vomiting (n = 175) | 19 (10.9) | 7 (25.0) | 12 (8.2) | **0.016*** |
| abdominal pain (n = 175) | 18 (10.3) | 18 (64.3) | 0 | **<0.001*** |
| diaphoresis (n = 175) | 15 (8.6) | 5 (17.9) | 10 (6.8) | 0.069 |
| conjunctival injection (n = 175) | 11 (6.3) | 4 (14.3) | 7 (4.8) | 0.078 |
| lymphadenopathy (n = 175) | 9 (5.1) | 2 (7.1) | 7 (4.8) | 0.638 |
| hepatomegaly (n = 175) | 2 (1.1) | 1 (3.6) | 1 (0.7) | 0.295 |
| splenomegaly (n = 175) | 2 (1.1) | 1 (3.6) | 1 (0.7) | 0.295 |
| **Hospitalization** | 49 (28.0) | 14 (50.0) | 35 (23.8) | **0.005*** |
| **Presence of leukopenia** | 123 (70.3) | 20 (71.4) | 103 (70.1) | 0.885 |
| **Presence of thrombocytopenia** | 131 (74.9) | 21 (75.0) | 110 (74.8) | 0.985 |
| **Presence of elevated liver enzymes** | 143 (81.1) | 25 (89.3) | 118 (81.9) | 0.421 |
| **Presence of lymphocytopenia (n = 66)** | 48 (72.3) | 10 (76.9) | 38 (71.7) | 1.000 |
| **White blood cell count** | | | | |
| Md (IQR) | 3.43 (2.55-5.19) | 3.21 (2.65-5.62) | 3.49 (2.5-5.15) | 0.799 |
| Min – Max | 1.21 -15.32 | 1.28-9.6. | 1.21-15.32 | |
| **Thrombocyte count** | | | | 0.511 |
| Md (IQR) | 127 (79.0-179) | 136.5 (44.25-173.8) | 127 (80-181) | 0.511 |
| Min – Max | 16-483 | 16-387 | 20-483 | |
| **Lymphocyte count (n = 66)** | | | | |
| Md (IQR) | 0.77 (0.45-1.26) | 0.60 (0.48–1.4) | 0.81 (0.47-1.39) | 0.227 |
| Min – Max | 0.17-5.12 | 0.28-2.58 | 0.17-5.12 | |
| **Duration of symptoms** | | | | |
| <7 days | 51 (33.8) | 6 (24.9) | 45 (35.7) | 0.463 |
| >7 days | 86 (49.1) | 17 (60.7) | 69 (46.9) | |

| | | | | |
|---|---|---|---|---|
| >14 days | 14 (8.0) | 2 (7.1) | 12 (8.2) | |
| **Duration of thrombocytopenia** | | | | |
| <7 days | 89 (61.4) | 13 (50.0) | 76 (63.9) | 0.212 |
| >7 days | 19 (13.1) | 6 (23.1) | 13 (10.9) | |
| none | 37 (25.5) | 7 (26.9) | 30 (25.2) | |
| **Duration of leukopenia** | | | | |
| <7 days | 85 (60.3) | 12 (44.4) | 73 (64.0) | **0.003*** |
| >7 days | 13 (9.2) | 7 (25.9) | 6 (5.3) | |
| None | 43 (30.5) | 8 (29.6) | 35 (30.7) | |
| **Duration of elevated hepatic enzymes** | | | | |
| <7 days | 46 (36.5) | 6 (35.3.) | 40 (36.7) | 1.000 |
| >7 days | 41 (32.5) | 6 (35.3) | 35 (32.1) | |
| >14 days | 15 (11.9) | 2 (11.8) | 13 (11.9) | |
| none | 24 (19.9) | 3 (17.6) | 21 (19.3) | |
